# Travel Anxiety, Risk Attitude and Travel Intentions towards “Travel Bubble” Destinations in Hong Kong: Effect of the Fear of COVID-19

**DOI:** 10.3390/ijerph17217859

**Published:** 2020-10-27

**Authors:** Jian Ming Luo, Chi Fung Lam

**Affiliations:** 1 Faculty of International Tourism and Management, City University of Macau, Macau, China; kenny.luo@connect.polyu.hk; 2 Department of Finance, The Chinese University of Hong Kong, Hong Kong, China

**Keywords:** COVID-19, fear, travel anxiety, risk attitude, intention

## Abstract

The impacts of COVID-19 are massive. Global tourism is one of the industries that is heavily affected. “Travel bubble”, a recent term initiated by travel operators, is a programme that allows tourists to travel to countries nearby without quarantine requirements. This study investigates the relationship amongst fear of COVID-19, travel anxiety, risk attitude and travel intention towards “travel bubble” destinations. Results show that fear of COVID-19, travel anxiety and risk attitude negatively impact travel intention. Furthermore, travel anxiety and risk attitude moderate the indirect impacts between fear of COVID-19 and travel intention. Future research and implications of practices are presented.

## 1. Introduction

World tourism has decreased by more than 80 percent since the outbreak of COVID-19 in 2019. In the first quarter of 2020, tourist arrivals dropped by more than 20 percent [1]. The COVID-19 outbreak has not only caused many hospitality-related industries, such as restaurants, bars and hotels, to shut down; many countries also shut down their borders, thus halting domestic and international travel. Hong Kong is no exception. Ever since the outbreak of COVID-19, 65 international airlines have reduced their flights by 95 percent. From January to July 2020, tourist arrivals decreased by over 90 percent [2].

Travel bubbles, also known as “Travel Bridges” or “Corona Corridors”, are considered a solution to the current pandemic, especially considering that the pandemic is expected to persist for a long time. The first “travel bubble” destination has been initiated by Estonia, Latvia and Lithuania. Citizens from these countries are allowed to travel freely (without quarantine requirements) within these countries [3]. Many Hong Kong tourism operators suggested the Hong Kong government to initiate this programme with countries or cities nearby where COVID-19 has been contained, such as Thailand, Japan, etc. [4,5]. However, Yetgin and Benligiray [6] argued that under this circumstance, economic and social pressures will cause many psychological and physiological diseases. This situation is worsened when people are surrounded by rumours and misleading false information about COVID-19 [7].

Previous research has shown that anxiety is a crucial determinant of behaviour [8]. For example, when people become more anxious about the virus, they tend to maintain a high level of personal hygiene, more social distancing and are more likely to get vaccinated when available [8]. In addition, they may overstock necessities, conduct unnecessary medical tests, or misinterpret their minor symptoms as signs of serious infection [9]. Anxiety is a mental state of tension and worry about the future [10] and high anxiety might act as a signal to avoid taking risks [11]. The outbreak of COVID-19 does not only make people anxious, but it also scares people. Therefore, understanding how this outbreak affects people’s mental health is important [12,13]. 

Although the desire to travel is closely related to the affective state of an individual, tourism researchers generally pay little attention to research on people’s feelings and emotions [14]. Some recent studies have explained how emotions can affect people’s behaviour [15,16]. More evidence can be found in studies of behaviour intentions [17]. Hence, emotion can affect how people decide their destinations and their behaviour [18]. Fear is a basic emotion [18]. Hence, the fear of the pandemic recently would affect people’s travel behaviour. COVID-19 does not only affect tourism but also the global economy. Researchers have studied the general effect of COVID-19 on tourism [19,20]. However, no studies have been conducted on travel behaviour, which includes the psychological effects of COVID-19. Therefore, the purpose of this study is to investigate the relationship between the fear of COVID-19, travel anxiety, attitude, and behaviour intention in “travel bubble” destinations.

## 2. Literature Review and Hypothesis Development

### 2.1. Fear of COVID-19

Emotion is hard to define because it is difficult to observe how emotion is developed, created and expressed [21]. The common consensus of the definition of emotion is that it is difficult to observe. Emotions involve a number of component processes encompassing subjective feelings, expressive motor behaviour, physiological arousal, cognitive appraisal and a behavioural tendency [22]. Many psychological emotion theorists agreed that emotion is not a simple phenomenon, and includes joy, acceptance, fear, surprise, sadness, anger, disgust and expectancy [23,24].

Since the outbreak, people have grown fearful of COVID-19. Fear, by general definition, is an emotion triggered by danger, pain or harm [25]. A viral outbreak, including COVID-19, could cause people to fear [26]. Researchers have studied fear and have developed many psychometric “fear scales” to measure the degree of “fear” of individuals. Recent studies, such as Ahorsu et al. [12], used these psychometric scales to measure the level of fear caused by COVID-19.

Regardless of the field of study, anxiety and fear are considered two different emotions [27]. Theoretically, fear is a primary emotion that is experienced by all human beings regardless of age, race and culture. Fear is an awareness of danger. Anxiety is the unpleasant feeling and physiological response when a person is scared [28]. COVID-19 has infected more than 10 million people worldwide; hence, people easily feel fear, panic and anxiety [12]. When there is a large amount of unverified, fake, or exaggerated information shared via social and online media, Rubin and Wessely [29] argued that such misinformation will increase the level of panic.

### 2.2. Travel Anxiety

Anxiety, loosely speaking, is an emotional response to stress, potential risks or actual risks. Gudykunst and Hammer [30] defined anxiety as fear of negative consequences. Dowling and Staelin [31] argued that when people buy something risky, the unknown consequence creates anxiety. McIntyre and Roggenbuck [32] extended this definition to include the feeling of being nervous, apprehensive, stressed, vulnerable, uncomfortable, disturbed, scared or panicked. Hullet and Witte [33] further included frustration and awkwardness in the definition.

Travelling to any destination involves risk and uncertainty; hence, people must evaluate a variety of factors, such as attributes of the products or destination, potential negative results, necessity and values. However, people have different evaluations of many products. For example, some people may consider one destination scary and dangerous, whereas others may regard the same destination as fun and exciting. Ahorsu et al. [12] found a positive relationship between the fear of COVID-19 and hospital anxiety. When people are constantly exposed to local and international news on fatalities and the infection rate of COVID-19, the degree of fear and anxiety increases. Hence, the level of life satisfaction decreases [34]. We formulate the following hypothesis.

**Hypothesis** **1 (H1).** *Fear of COVID-19 positively affects travel anxiety towards “travel bubble” destinations*.

### 2.3. Risk Attitudes

Attitude is a stable and persistent psychological construct that can effectively affect and predict human behaviour [35]. Risk attitude, as the name suggests, is an attitude towards risk. It is a mindset on risk-taking behaviour under an uncertain or risky environment [36]. People usually act rationally under risk or uncertain circumstances. They may hedge or mitigate the risk entirely or partially [37]. Although complete avoidance of risk is sometimes possible, this option is less likely chosen [37]. Instead, people usually take measures, such as creating back-up plans or flexible plans according to the nature of the risk [38]. Risk perception or the perception of risk is important to consumers’ decisions and judgement [39]. Risk perception is a complex perception formed by an individual on the basis of the impact of the negative consequence and the environment. Researchers argued that people tend to optimise their risk-taking behaviour by balancing the expected benefit and loss [40], whereas other researchers argued that people form their perception from individual experiences and environmental circumstances, such as media reports [41,42]. Recently, Xie, Huang, Li, & Zhu [43] found that COVID-19 affects people’s perception of and attitude towards food. Thus, we propose the following hypotheses.

**Hypothesis** **2 (H2).** *Fear of COVID-19 positively affects risk attitude towards “travel bubble” destinations*.

**Hypothesis** **3 (H3).** *Travel anxiety positively affects risk attitude towards “travel bubble” destinations*.

### 2.4. Travel Intention

Travel intention is defined as one’s desire or intention to travel. Travel intention has two sources: personal and information source. In the process of forming perception, information sources are relatively important [44]. In addition to personal and information source, risk and safety are important factors that determine travel intention. Risk is associated with anxiety because of what may happen during a trip. For example, the possibility of terrorism in a destination will commonly form a perception of danger. This perception will result in a corresponding decision. Under such circumstances, people will tend to choose less risky destinations [45,46].

When a destination is viewed as “unsafe”, people may form a negative perception [47]. This form of perception is developed via information sources, such as news from mainstream and social media [48]. For example, when mainstream media focuses on the number of infected people, the number of deaths, the number of stores that have closed and the businesses that have gone bankrupt due to COVID-19, people begin to worry about their jobs. People will grow anxious and their perceived level of safety in a destination decreases; hence, travel intention is reduced [49,50]. Therefore, we propose the following hypotheses.

**Hypothesis** **4 (H4).** *Travel anxiety negatively affects travel intention towards “travel bubble” destinations*.

**Hypothesis** **5 (H5).** *Risk attitude negatively affects travel intention towards “travel bubble” destinations*.

**Hypothesis** **6 (H6).** *Fear of COVID-19 negatively affects travel intention towards “travel bubble” destinations*.

## 3. Method

This study uses measurement scales from existing research to develop the questionnaire. Previous studies have shown that these measurement scales are valid and significant. This study uses a 5-point Likert scale, where 1 means “strongly disagree” and 5 means “strongly agree”. SPSS (IBM Corp., Armonk, NY, USA) and AMOS 22.0 (IBM Corp., Armonk, NY, USA) are used to process the data and estimate the structural equations. The description of the scale is as follows:

Seven items measure *Fear of COVID*, and these items were developed by Ahorsu et al. [12] and Satici et al. [34]. A sample item is “I am afraid of losing my life because of COVID-19”.

Six items measure *Travel Anxiety*, and these items were developed by Reisinger and Mavondo [49] and Wachyuni and Kusumaningrum [50]. A sample item is “I will panic when I travel during the COVID-19 pandemic”.

Three items measure *Risk Attitude*, and these items were adopted from Zhu and Deng [46]. A sample item is “I will not eat with local friends and relatives after their trip to a travel bubble destination”.

Three items measure *Travel Intention*, and these items were adopted from Reisinger and Mavondo [49] and Zhu and Deng [46]. A sample item is “I prefer to travel to ‘travel bubble’ destinations over other forms of tourism”.

All items were back-translated in Chinese, and bilingual tourism scholars verified the accuracy of the translation. Moreover, marketing and tourism scholars checked the validity of the content. We further conducted a pilot test before the actual survey. Fifteen individuals were invited to participate in the pilot test. Changes were made on the basis of their opinions. In the final version, 19 items were included in the measurement scales. The remaining parts of the questionnaire collected the demographic information of participants.

The targets are Hong Kong residents with knowledge about “travel bubble” destinations. The screening questions in the questionnaire will assure the identity of the respondents. Since the data was collected during the pandemic when social distancing should be imposed, therefore, an online survey was the best option [51]. Since 06 July 2020, news from online and traditional media has reported that there are 8 to 10 countries to be included in the travel bubble arrangements [4]. This study adopts convenient sampling. Online questionnaire with a web link was sent via personal contacts in social media. The questionnaire was initially distributed to the researchers’ personal network on WhatsApp and WeChat in Hong Kong. No incentive scheme was provided. Respondents could forward the survey to their friends and relatives in Hong Kong from July 6 to Aug 16, 2020. A total of 303 questionnaires were usable.

## 4. Results

### 4.1. Respondent Profiles

Gender distribution is roughly equal. Several respondents are single/divorced/widowed, have an undergraduate degree and aged 18 to 45 years old. Most of them are working and have a monthly income ranging from HKD 10,001 to 50,000 per month (see Table 1).

### 4.2. Measurement Model

SPSS20 and AMOS22 are used to perform confirmatory factor analysis (CFA). The results show the CFA is valid in terms of reliability, convergent validity and discriminant validity. Table 2 shows that the Cronbach’s alphas of all coefficients are between 0.904 and 0.919. This result satisfies the minimum requirement [52]. The result of convergent validity is also satisfactory. All factor loadings are significantly different from zero, ranging from 0.631 to 0.928. The composite reliability of each construct is higher than 0.5. According to Fornell and Larcker [53], discriminant validity can be examined by the difference of the average variance and the squared root correlation (see Table 3). The measurement model is valid, that is, the standardised chi-squared is 2.348, Comparative Fit Index (CFI) is 0.954, Tucker–Lewis Index (TLI) is 0.946, Goodness of Fit Index (GFI) is 0.886 and the acceptable range of root-mean-square error of approximation(RMSEA) is 0.067. These numbers indicate that the model is acceptable [54].

### 4.3. Structural Model Testing

**Figure 1:**
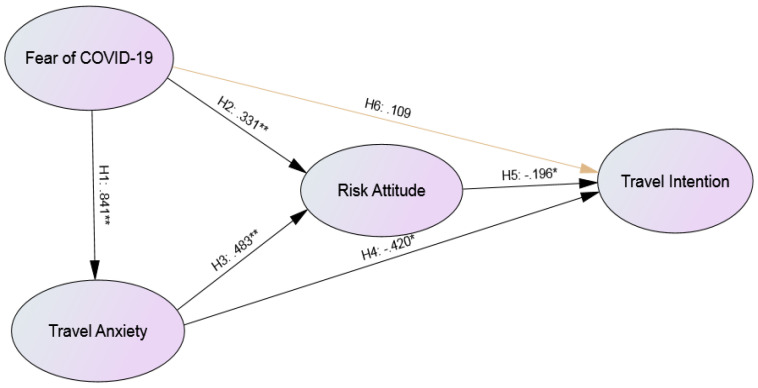
Results of the Hypotheses (Note: ** *p* value < 0.01; * *p* value < 0.05).

This study uses structural equation model (SEM) and maximum likelihood to examine the relationship amongst fear of COVID-19, travel anxiety, risk attitude and travel intention towards “travel bubble” destinations. The chi-squared value is 342.795 (99% significance). Other indicators confirm that the model is acceptable and the data are consistent with the model. The hypothesis paths are hence tested. Table 4 and Figure 1 show the results of the hypothesis testing. The first five hypotheses are significant, namely, H1 (*t* = 12.961, *p* < 0.01), H2 (*t* = 3.448, *p* < 0.01), H3 (*t* = 4.944, *p* < 0.01), H4 (*t* = −3.111, *p* < 0.05) and H5 (*t* = −1.981, *p* < 0.05). However, H6 is non-significant. This result indicates no evidence to suggest a relationship between fear of COVID-19 and travel intention.

The SEM results also show that the indirect effect of fear of COVID-19, connected by travel anxiety and risk attitude, towards travel intention was −0.532 (Table 5). Bootstrapping is employed to investigate the significance of the mediating effect between risk attitude and travel anxiety. After 1000 resamplings, the 95% confidence interval is [−0.782, −0.230]. The interval does not contain zero, which shows that the meditating effect is significant. This result suggests that fear of COVID-19 influenced travel intention through travel anxiety and risk attitude.

## 5. Conclusions

The COVID-19 outbreak created much fear in human society, especially among marginalized and vulnerable people [55]. One of the reasons of such an outbreak is the infection rate of COVID-19. Therefore, many countries utilized different quarantine procedures to reduce the number of imported cases. Under this condition, tourism is severely affected [56]. The results of this study show that people in Hong Kong are increasingly aware of safety in travel. The results revealed important elements that increase travel intention. Although fear of COVID-19 directly affects travel anxiety and risk attitude, travel anxiety and risk attitude have direct negative effects on travel intention. However, no direct relationship exists between fear of COVID-19 and travel intention. In conclusion, Hypotheses 1 to 5 are supported, and Hypothesis 6 is not.

The results confirm the existing literature. Risk and security are some of the key concerns of tourists [49]. When anxiety and risk attitude decrease, travel intention increases. The results support the findings of previous studies [45,46,49,57]. This study introduced travel anxiety and risk attitude as mediating variables between fear of COVID-19 and travel intention. While fear of COVID-19 positively affects both travel anxiety and risk attitude (H1 and H2), anxiety also positively affects risk attitude (H3). The effects of travel anxiety and risk attitude on travel intention are negative and significant (H4 and H5). But the effect of fear of COVID-19 on travel intention is not significant (H6). This means that there is no evidence to suggest that fear of disease reduces people’s travel intentions. One explanation of this insignificant result is that COVID-19 is present worldwide; even when people are afraid of COVID-19, the differences between the risks in their hometown and that of a destination may not vary significantly. However, the fear of COVID-19 significantly impacts people’s travel anxiety and risk attitude, ultimately making them afraid to travel.

## 6. Implications and Future Research

### 6.1. Theoretical Implications

This study provides some major contributions to tourism literature. First, this study introduces a construct, the fear of COVID-19, into a model. Emotion, which affects people’s perceptions and actions, is an important factor that affects human behaviour [58]. This factor is important not only because of its ability to affect human behaviour, but also because of its persistent evolution over time [59]. When an event happens, people will first evaluate it on the basis of their individual experiences, culture and religion. Emotion will then be developed on the basis of this evaluation, and people will respond accordingly. Therefore, each emotion can be explained by using psychological emotion theory [60]. This result can explain how emotion affects people’s travel decision from the psychological perspective. Second, this study assesses the indirect impact of the fear of COVID-19 by introducing several mediators, travel anxiety, risk attitudes, and travel intentions. Our results show that fear of COVID-19 affects travel anxiety, risk attitudes, and travel intentions differently. We verified the mediating effects of these three variables. Travel anxiety and risk attitudes are two important mediating variables. This contributes to the tourism literature on tourist behaviours.

Lastly, our results contribute to the destination management literature on factors of outbound tourism. Hong Kong is one of the largest outbound tourist generators in the Asia–Pacific region [61]. Many previous studies of Hong Kong residents’ destination choices focus on trip characteristics, socio-demographics and travel motivations [62]. None of the previous studies examined “travel bubble” destinations as a destination choice.

### 6.2. Managerial Implications

From the practical point of view, this study provides a good understanding of how fear of COVID-19 affects travel intention via anxiety and risk attitude. This knowledge is crucial for marketing a destination. One of the findings of this study is that the fear of diseases does not deteriorate people’s travel intention while anxiety and risk attitude do. Therefore, tourism practitioners, destination marketing organization (DMO)s and governments should focus on reducing people’s anxiety and providing a positive risk attitude. Tourism practitioners can increase the appeal of a destination by reducing the perception of risk. In particular, service providers need to have guidelines on cleanliness and services to make sure that guests feel safe. Hotel, airlines and transportation companies should have customized services and stringent safety processes to let tourists feel comfortable within travel. Alternatively, tourism practitioners can explain their contingency plan to tourists more clearly, such that tourists can perceive risks as manageable and under control.

DMOs and the government should use different methods to provide sufficient information to satisfy people’s demand for safety, especially for those who have low risk tolerance [46]. Information can be distributed through many resources, such as advertisements on public transportation, social media and health information centres. In addition, these measures to enhance public safety will improve people’s perception of safety. Perception of risk is usually affected by local tour operators, guides, news and social media [45]. Although local operators and guides may exaggerate the degree of safety, news and social media may exaggerate the degree of riskiness. Both forms of misguidance may lead to unreasonable concerns. When people are too optimistic, they may become careless, thus increasing the chance of infection. However, when people are too pessimistic, they become anxious. To reduce travel anxiety, tourism practitioners can provide more information about the current risk level of a destination. Alternatively, they can repackage their products to downplay high-risk attractions or activities [49]. Tourists should also be encouraged to obtain information from other resources. Professional and unbiased tour operators can provide accurate information, which reduces the level of misperception and increases the level of security. As a result, the level of anxiety is reduced.

### 6.3. Limitations and Future Research

Since tourism is one of the pillar industries in Hong Kong, therefore, the “travel bubble” is an important tool to recover the economy. The eastern and western countries have different cultures and attitudes to the “travel bubble” destinations. Western people have more carefree attitudes and intentions in travelling during the pandemic, and it is worth comparing the results. A comparative study of the methodologies used in the study in other geographical areas and naturally related to the same theme would have been important. Future research can examine the hypotheses with random samples collected from different countries. This approach would reduce the bias of culture, ethnicity and geography. This study only examined a few constructs, but other constructs such as personality, lifestyle and motivation could be important. Thus, future researchers may consider incorporating other constructs to enrich the model. Finally, given that the COVID-19 outbreak eliminates face-to-face interviews, all surveys were conducted online. Hence, the sample size is limited. Therefore, researchers could increase the sample size to increase the representativeness of the study.

## Figures and Tables

**Table 1:** The Demographic Profile of Participants.

Variable		Frequency (*n* = 303)	%
Gender	Male	165	54.5
	Female	138	45.5
Marital Status	Married	129	42.6
	Single/Divorced/Widowed	174	57.4
Age	18–25	86	28.4
	26–35	59	19.5
	36–45	68	22.4
	46–55	61	20.1
	55 or above	29	9.6
Education	High school or below	43	14.2
	Diploma	63	20.8
	Undergraduates	124	40.9
	Graduates or above	73	24.1
Occupation	Working	208	68.6
	Student	50	16.5
	Housewife	17	5.6
	Retired	23	7.6
	Others	5	1.7
Income	HKD 10,000 or below	46	15.2
	HKD10,001–30,000	100	33.0
	HKD30,001–50,000	81	26.7
	HKD50,001 or above	76	25.1

**Table 2:** Results of Confirmatory Factor Analysis.

Latent Variable	Measured Item	Standardized Loading	Cronbach’s Alpha	Component Reliability (CR)	Average Variance Extracted (AVE)
Fear of COVID-19	1. I am most afraid of the novel coronavirus	0.794	0.904	0.906	0.582
	2. It makes me uncomfortable to think about novel coronavirus	0.732			
	3. My hands become sweaty when I think about COVID-19	0.787			
	4. I am afraid of losing my life because of COVID-19	0.798			
	5. When watching news and stories about novel coronavirus on social media or any other media (i.e., TV, Radio), I become nervous or anxious	0.631			
	6. I cannot sleep because I am worried about getting the novel coronavirus	0.81			
	7. My heart races or palpitates when I think about getting COVID-19	0.771			
Travel Anxiety	8. I feel uncomfortable after thinking of going on a tour during a pandemic	0.794	0.919	0.920	0.656
	9. I feel that my body is not fit after planning tourism activities during the pandemic	0.838			
	10. I was afraid to go on a tour during the pandemic	0.82			
	11. I will panic when I travel during the COVID-19	0.823			
	12. I sweat after deciding to travel during a pandemic	0.78			
	13. I feel an irregular heartbeat when I think of going on a tour during the pandemic	0.803			
Risk Attitude	14. I cannot accept going to travel to the “travel bubble” destinations with family and friends	0.865	0.909	0.906	0.762
	15. I cannot accept that local friends and relatives travel to the travel bubble destinations	0.928			
	16. I will not eat with local friends and relatives after their trip to the travel bubble destination	0.823			
Travel Intention	17. I would like to travel to the “travel bubble” destination for some time in the future	0.902	0.909	0.910	0.771
	18. I prefer to travel to the “travel bubble” destination compared with other forms of tourism	0.825			
	19. I will recommend the “travel bubble” destination to relative or friends	0.905			

**Table 3:** Latent Variable Correlation Coefficients.

Latent Variable	Number of Items	Fear of COVID-19	Travel Anxiety	Risk Attitude	Travel Intention
Fear of COVID-19	7	0.763 *			
Travel Anxiety	6	0.402	0.810 *		
Risk Attitude	3	0.465	0.568	0.873 *	
Travel Intention	3	−0.214	−0.312	−0.375	0.878 *

* The root square of the average variance extracted for each construct

**Table 4:** Direct Path for the Structural Model.

Hypothesis	Path	Standard Coefficient	*t*-Value	*p*-Value	Decision
H1	Fear of COVID-19 → Travel Anxiety	0.841	12.961	0.000	Accept
H2	Fear of COVID-19 → Risk Attitude	0.331	3.448	0.000	Accept
H3	Travel Anxiety → Risk Attitude	0.483	4.944	0.000	Accept
H4	Travel Anxiety → Travel Intention	−0.420	−3.111	0.002	Accept
H5	Risk Attitude → Travel Intention	−0.196	−1.981	0.048	Accept
H6	Fear of COVID-19 → Travel Intention	0.109	0.855	0.393	Reject

**Table 5:** Results for Mediation Tests.

Causal Relationships	Direct Effect	Indirect Effect	Total Effect
Fear of COVID-19 → Travel Anxiety	0.996	-	0.996
Fear of COVID-19 → Risk Attitude	0.518	0.635	1.153
Travel Anxiety → Risk Attitude	0.637	-	0.637
Travel Anxiety → Travel Intention	−0.486	−0.109	−0.595
Risk Attitude → Travel Intention	−0.171	-	−0.171
Fear of COVID-19 → Travel Intention	0.149	−0.681	−0.532
